# Prediction of sensory attributes in winemaking grapes by on-line near-infrared spectroscopy based on selected volatile aroma compounds

**DOI:** 10.1007/s00216-023-04549-2

**Published:** 2023-01-27

**Authors:** Jana Gehlken, Martin Pour Nikfardjam, Christian Zörb

**Affiliations:** 1 State Research Institute for Viticulture and Pomiculture, Traubenplatz 5, 74189 Weinsberg, Germany; 2 Institute of Crop Science, Quality of Plant Products and Viticulture (340E), University of Hohenheim, Emil-Wolff-Straße 25, 70593 Stuttgart, Germany

**Keywords:** Near-infrared spectroscopy, Grape tasting, Aroma compounds, Sensory attributes, Calibration

## Abstract

Aroma represents an important quality aspect for wine. The aroma of different grapes and wines is formed by the varying composition and concentrations of numerous aroma compounds, which result in different sensory impressions. The analysis of aroma compounds is usually complex and time-consuming, which requires the development of rapid alternative methods. In this study, grape mash samples were examined for aroma compounds, which were released under tasting conditions. A selection of the determined aroma compounds was grouped according to their sensory characteristics and calibration models were developed for the determination of sensory attributes by near-infrared (NIR) spectroscopy. The calibration models for the selected sensory attributes “fruity,” “green,” “floral” and “microbiological” showed very high prediction accuracies (0.979 < *R*^2^_C_ < 0.996). Moreover, four different grape model solutions, whose compositions were based on the results from GC–MS-based analysis of the grape mash samples, were examined in a sensory evaluation. Despite large variation of the single values, the averaged values of the given scores for intensity of odour and taste showed differences between the model solutions for most of the evaluated sensory attributes. Sensory analysis remains essential for the evaluation of the overall aroma; however, NIR spectroscopy can be used as an additional and more objective method for the estimation of possible desired or undesired flavour nuances of grape mash and the quality of the resulting wine.

**Graphical Abstract:**

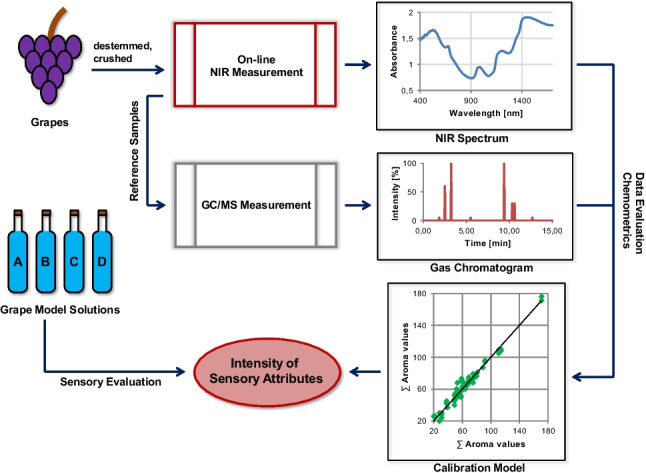

## Introduction

The composition of aroma is a very complex issue. Only in few foods the aroma is characterised by one single compound (a so-called character impact compound), e.g. vanilla by vanillin or raspberries by 4-(4-hydroxyphenyl)butan-2-one (raspberry ketone), while usually the composition of various compounds is responsible for the overall aroma. The aroma of most fruits, such as grapes, is usually formed by 200 to 400 different compounds [1]. Every aroma compound is related to specific odour and/or taste characteristics and, depending on its concentration as well as on its odour and taste thresholds, contributes to the characteristic aroma. Some grape varieties are described as quite “neutral” and most aroma compounds originate from fermentation and ageing processes of wine; however, other varieties produce more “aromatic” grapes. The aroma of wines made from these varieties is influenced considerably by compounds originating from the grapes, such as terpenoids. Although other varieties produce “neutral” grapes with little to no characteristic varietal aroma, wines made from these grapes show recognisable flavour characteristics highly depending on the variety [2, 3]. Therefore, certain flavour characteristics are expected for certain wines, which means that aroma represents an important quality aspect. However, quality is an individual and subjective response depending on the judging person and, therefore, may often be influenced by personal preferences and experiences. For this reason, high quality is often associated with “like,” while many quality assessments are to test whether certain expectations and/or specifications are met and therefore should be completely objective [2]. Objective results are usually obtained by instrumental analysis, which is very complex due to the high number and mostly low concentrations of aroma compounds in foods.

The most widespread method for the analysis of aroma compounds is gas chromatography, often coupled with mass spectrometry (GC–MS), which allows the simultaneous detection and quantification of manifold compounds. Moreover, GC–MS analysis offers high sensitivity, which is necessary because aroma compounds are often present in trace amounts (ppb or even lower). Although high precision and accuracy can be reached, various disadvantages come along, such as the requirement of expensive instrumentation and high-purity chemicals. Furthermore, sample preparation, analysis and evaluation of the results are very time-consuming and require experienced users. Even though many compounds can be measured instrumentally, the final aroma cannot generally be predicted from these results, because reliable correlations between chemical structures and perceived aroma impression are still widely unexplored [1, 2]. Additionally, numerous compounds may be detected in GC–MS analysis, which do not contribute to the aroma of the analysed sample at all, because the determined concentration lies below odour and/or flavour threshold. Therefore, the presence and intensity of certain odour and flavour nuances are usually examined during sensory analysis, which may provide more reliable results than instrumental analysis, because pleasure is an important factor of tasting [2]. However, several shortcomings have to be considered. Odour and flavour perception is influenced by numerous physical (e.g. temperature), chemical (e.g. pH value), biological (e.g. saliva production and composition) or psychological (e.g. tiredness) factors. Odour and flavour thresholds often vary between different persons and may also fluctuate considerably for the same person depending on e.g. time of day. Moreover, personal preferences may have an effect on the results, and the description of odour and flavour notes can be very difficult without the appropriate vocabulary [2, 4]. Therefore, numerous rules need to be considered to obtain reliable results in sensory evaluation. To avoid any bias caused by the environment, sensory analyses require specially equipped tasting rooms. Furthermore, the participants need extensive training to minimise the influence of individual factors, such as chewing and swallowing techniques [4], and personal preferences, and to acquire the vocabulary for description. However, even with trained panellists, the results of sensory analyses still remain subjective due to factors, which play an important role in aroma release and perception and cannot be standardised, such as salivary composition [2, 4]. Moreover, the collection and evaluation of data from sensory analysis is very time-consuming compared to instrumental analysis. All in all, sophisticated analytical methods are still not able to provide sufficiently reliable results, while sensory analysis means a higher effort. Although sensory analysis is still essential for the evaluation of quality, further development of instrumental methods for rapid, easy and objective analysis is required.

Recently, alternative methods for classical wet-lab analyses become increasingly important, which results in increasing interest in spectroscopic methods, such as NIR spectroscopy. The use of NIR spectroscopy not only is considerably cheaper and easier to use than common analytical methods (e.g. GC–MS), but also allows simultaneous determination of various compounds [5]. Moreover, only minimal or no sample preparation is necessary and the measurement can be integrated into an existing process, which saves the time otherwise needed for additional sampling. Due to the ongoing development of spectrometers and chemometric techniques, NIR spectroscopy provides a suitable alternative for time-consuming and expensive analytical methods. On-line NIR measurements have already been successfully used under industrial conditions to evaluate the quality of grape mashes [6]. The determination of sensory attributes by NIR spectroscopy has not been examined in grapes yet, but several studies with wines showed partly excellent results. Although some relationships could be identified between NIR spectra and certain sensory properties, exact knowledge about the responsible chemical compounds is still missing [7–10]. NIR spectroscopy has also been applied successfully to examine flavour parameters of citrus fruits. However, in this study, certain chemical compounds have been defined as flavour parameters [11]. In grapes, NIR spectroscopy has been used successfully for the determination of glycosidic aroma compounds in grape juice [12], Tannat grapes [13, 14] and grapes exposed to bushfires [15].

The aims of this study were to develop calibration models for the determination of sensory attributes in grapes by NIR spectroscopy and to compare the results to those from a sensory evaluation. For the calculation of calibration models, the sensory attributes were based on selected aroma compounds. This way, an objective method for the evaluation of grape aroma quality by on-line NIR spectroscopy directly upon receival of the grapes at the winery should be provided to enable a comparison of the delivered grapes according to their aroma quality in addition to usual quality parameters. To the best of our knowledge, this is the first approach of determination of sensory attributes in grapes by on-line NIR spectroscopy.

## Materials and methods

### Samples

Grape mash samples were provided by Lauffener Weingärtner eG from the vintage 2019 (Table 1). To ensure that the sample material for reference analysis was identical to the sample material analysed by the NIR sensor, samples were taken directly from the grape reception line at the moment of NIR measurement. The samples were filled into plastic bottles (500 mL) containing 250 mg/kg sodium azide for preservation. Samples were stored deep-frozen (− 20 °C) until analysis.

**Table 1:** Numbers of grape mash samples from different grape varieties taken from the vintage 2019 at Lauffener Weingärtner eG

Grape variety	Number of samples
Cabernet Dorsa	2
Gewürztraminer	1
Grauburgunder	1
Kerner	1
Lemberger	10
Riesling	5
Samtrot	6
Schwarzriesling	3
Spätburgunder	5
Trollinger	8

### NIR measurements

An X-Three V3 sensor with an InGaAs detector (NIR range) and a Si detector (visible range) (Büchi NIR-Online, Walldorf/Germany) was integrated into the grape reception line of the winery. For data acquisition, the SX-Center software (Version 2.13.1000.453, Büchi NIR-Online, Walldorf/Germany) was used. Spectra were collected in diffuse reflectance mode in the wavelength range between 400 and 1700 nm with intervals of 5 nm. During the measurement (15 s), a total number of 300 spectra (20 spectra/s) was acquired and then averaged.

### Reference analysis

#### GC–MS analysis of aroma compounds in grape mash samples

Aroma compounds should be analysed under conditions as similar as possible to those in the mouth. Sample preparation and measurement conditions were chosen on the basis of previous examinations [16]. Each sample was prepared directly before analysis to ensure equal measurement conditions and to avoid loss of volatile compounds or artefact formation. Sample material was thawed overnight at ambient temperature and homogenised with a commercial hand blender (ESGE-Zauberstab 2007–5, Unold AG, Hockenheim/Germany). An amount of 5 g of the homogenised sample material was weighed into a headspace vial (20 mL, clear glass/crimp top/round bottom, neoLab Migge GmbH, Heidelberg/Germany) and covered with a cap (Aluminium, PTFE/Silicone septum, Perkin Elmer, Rodgau/Germany). Ten minutes after homogenisation, the vial was shaken carefully to eliminate possible sedimentation of solid components. As an internal standard, 10 µL of a solution of 2-heptanone (analytical grade, Frey & Lau GmbH, Henstedt-Ulzburg/Germany) in HPLC grade water (*c* = 25 mg/L) was added. The cap was closed and the vial was placed into the headspace sampler.

For GC–MS analysis, a Turbomatrix 40 Trap Headspace Sampler, a Clarus 600 Gas Chromatograph and a Clarus 600C Mass Spectrometer (Perkin Elmer, Rodgau/Germany) were used with an Elite-624 capillary column (30 m length, 0.25 mm inner diameter (i.d.), 1.4 µm film thickness; Perkin Elmer, Rodgau/Germany) and helium (BIP grade; Tyczka Industrie-Gase GmbH, Mannheim/Germany) as carrier gas. The exact settings of the headspace sampler are shown in Table 2. The GC oven temperature was set at 40 °C and held for 5 min, then raised to 220 °C with a rate of 20 °C/min and held again for 10 min. Mass spectrometry was carried out in electron ionisation (EI^+^) mode (70 eV) scanning the mass range between 40.00 and 200.00 m/*z*. Data was acquired and analysed with the TurboMass software (Version 5.4.2). The detected compounds were identified by comparing the mass spectra to the NIST library (spectra library: NIST/EPANIH mass spectral library; search program: NIST MS search, Ver. 2.0) and, if available, to reference substances. Additionally, the retention times of the detected compounds were compared to reference substances, if available. For quantification, reference substances were dissolved in HPLC grade water and measured in three different concentrations to calculate calibration curves. Reference substances were provided by Alfa Aesar (Thermo Fisher GmbH, Kandel/Germany), Frey & Lau (Frey & Lau GmbH, Henstedt-Ulzburg/Germany), Sigma-Aldrich (Merck KGaA, Darmstadt/Germany) and VWR (VWR International GmbH, Darmstadt/Germany) with a purity of at least 95%.

**Table 2:** Headspace parameters for GC–MS analysis

Temperature [°C]		Timing [min]		Option		PPC [psi]	
Oven Needle Transfer Trap Hi Trap Lo	37 50 200 280 40	Thermo Dry purge Desorb Trap hold GC cycle	5.0 5.0 2.5 10.0 25.0	Operating mode Dry purge Outlet split	Trap Yes Yes	Column^a^ Vial Desorb	20.0 25.0 20.0

^a^Equivalent to a column flow rate of 2.5 mL/min (40 °C)

#### Sensory evaluation of grape model solutions

Due to the use of sodium azide for preservation of the grape mash samples, tasting was not possible. Instead, four different aqueous model solutions were prepared for sensory evaluation based on the concentrations of aroma compounds in various measured grape mash samples. Commercially available glucose and fructose from the supermarket and tartaric acid from the pharmacy were used. Ethanol was used in drinking quality (BrüggemannAlcohol Heilbronn GmbH, Heilbronn/Germany). The other aroma compounds were provided by Sigma-Aldrich (Merck KgaA, Darmstadt/Germany) in food grade quality. The exact compositions of the four solutions are listed in Table 3.

**Table 3:** Composition of the model solutions for sensory evaluation

Compound	Solution A	Solution B	Solution C	Solution D
Glucose [g/L]^a^	72.0	72.0	72.0	72.0
Fructose [g/L]^a^	74.0	74.0	74.0	74.0
Tartaric acid [g/L]^a^	5.3	5.3	5.3	5.3
Acetaldehyde [µg/L]	20,000.0	30,000.0	7000.0	40,000.0
Ethanol [µg/L]	300,000.0	400,000.0	200,000.0	400,000.0
Methyl acetate [µg/L]	1000.0	900.0	30.0	150.0
Isobutanal [µg/L]	40.0	50.0	10.0	250.0
3-Methylbutanol [µg/L]	800.0	2000.0	90.0	900.0
Hexanal [µg/L]	4000.0	10,000.0	5000.0	7000.0
Ethyl 3-methylbutanoate [µg/L]	1.0	1.0	-	-
2-Hexenal [µg/L]	1000.0	6000.0	2000.0	2000.0
Linalool [µg/L]	-	-	40.0	-

^a^Concentrations adapted from [17]

Sensory evaluation of the model solutions was carried out according to the rapid method CATA (check all that apply), which originates from the work of C. H. Coomb in 1964. A list of attributes is given, from which the participants choose all the ones applying to the sensory properties of the sample [18]. In our study, the version RATA (rate all that apply) was selected, where the intensity of the attributes is rated additionally to increase sample discrimination [19]. Seven attributes were given (fruity/citrus, fruity/apple, fruity/banana, vegetative/grass, floral, alcoholic, pungent), which were chosen on the basis of the aroma descriptions of the used aroma compounds [20] and the wine aroma wheel [21].

The tasting session was carried out in the tasting room of the State Research Institute for Viticulture and Pomiculture in Weinsberg with constant light and temperature conditions. Sensory analysis of the model solutions was performed by a panel of 30 participants (20 males and 10 females) with varying experience in tasting. The model solutions were rated on a four-point scale (1: not/barely detected, 2: low intensity, 3: medium intensity, 4: high intensity). During a first run, only the odour intensity of the sensory attributes was checked and rated for the model solutions, while in a second run, the solutions were tasted and the intensity of the attributes was rated.

### Chemometrics and data analysis

The SX-Plus software (Version 2.13.1000.453, Büchi NIR-Online, Walldorf/Germany) was used for processing spectral and reference data. Calibration models were calculated with the XLS regression method, which consists of partial least squares (PLS) regression and the first derivative of the spectra. The number of latent variables (LV) was limited to a maximum of 15. All calibration models were calculated without spectral pretreatment, after normalisation by standard normative variate transformation (SNVT) and after normalisation by multiplicative scatter correction (MSC). Segmented cross-validation (*S* = 5) was chosen for the validation of the models due to the small size of the dataset. Model performance was evaluated based on the correlation coefficients of calibration (*R*^2^_C_) and cross-validation (*R*^2^_CV_), the standard errors of calibration (SEC) and cross-validation (SECV) and the residual predictive deviation (RPD). The overall smallest value for the SECV was chosen to select the spectral pretreatment and the number of LV for the calibration models.

Statistical evaluation of the results from the sensory evaluation was carried out using Origin 2020 software (Version 9.7.0.185, OriginLab Corporation, Northampton/USA). Principal component analyses (PCAs) were executed to evaluate possible correlations for selected aroma compounds and to display the results of the sensory evaluation. Moreover, one-way analysis of variance (ANOVA) was performed to investigate the differences among the scores given to the model solutions in the sensory evaluation.

## Results and discussion

### Calibration models of grape aroma attributes

Fifty-one compounds have been detected in the examined grape mash samples (Table 4).

**Table 4:** Detected aroma compounds in the examined grape mash samples

Compound	Retention time [min]	*m*/*z* qualitative	*m*/*z* quantitative	Concentration range [µg/kg]	Taste threshold in water [µg/L]
Acetaldehyde	1.77	44	44	3.2–40.1^b^	22^d,e^
Pentane^a^	2.24	43	43	-	> 500,000 (fish)^e^
Ethanol	2.47	45	45	102.6–431.0^b^	10,000^e^
Methyl acetate	3.21	43, 74	74	18.6–1058.8	50 (in beer)^e^
2-Methyl-1-propanal (Isobutanal)	3.97	43, 72	72	7.4–293.9	0.4^e^
1-Propanol	4.59	42, 59	59	309.9–2117.3	7000^e^
2,3-Butanedione (Diacetyl)	5.20	43, 86	86	29.3–224.1	5.4^d,e^
Ethyl actetate	5.44	43, 61	61	92.7–12,784.3	3000^d,e^
2-Butanol	5.71	45, 59	59	43.9–318.9	5100^e^
2-Methyl-1-propanol (Isobutanol)	6.52	43, 74	74	68.5–3000.2	8000^e^
3-Methylbutanal	6.74	44, 58, 71	58	1.9–35.7	170^d,e^
Acetic acid	6.83	43, 45, 60	60	8.2–363.8^b^	54,000^d,e^
2-Methylbutanal	6.89	41, 57	57	1.0–36.6	0.8^e^
2-Ethylfuran	7.23	53, 81, 96	81	0.03–0.36	-
1-Butanol	7.30	41, 43, 56	56	11.0–182.0	500^e^
1-Penten-3-one	7.48	55, 84	55	1.5–17.7	1^e^
2-Pentanone	7.53	43, 86	86	2.4–3.8	860,000^e^
1-Penten-3-ol	7.59	57	57	14.7–131.4	3^e^
Pentanal	7.63	44, 57, 58	57	1.4–16.8	70^d,e^
Ethyl propanoate	7.67	45, 57	57	0.2–0.8	4^e^
Propyl acetate	7.74	43, 61, 73	61	0.7–9.8	800^e^
2,4,5-Trimethyl-1,3-dioxolan^a^	7.82	43, 55, 72	55	-	-
Ethyl isobutanoate	8.46	43, 71	71	0.2–2.3	0.03^e^
3-Methylbutan-1-ol	8.55	42, 55, 70	55	74.9–2816.8	100^e^
2-Methylbutan-1-ol	8.60	41, 57, 70	57	26.0–883.5	5500^e^
Isobutyl acetate	8.75	43, 56, 73	56	0.7–16.3	300^e^
1-Pentanol	9.04	42, 55, 57, 70	55	1.7–5.2	4500^e^
Ethyl butanoate	9.11	43, 71, 88	71	0.3–1.0	0.13^d,e^
2-Penten-1-ol^a^	9.16	41, 57, 68	57	-	-
Hexanal	9.34	41, 44, 56	56	2.0–10.8^b^	3.7^d,e^
Ethyl 3-methylbutanoate	9.83	57, 60, 85, 88	88	0.1–1.1	0.01^d^
3-Methylbutyl acetate	10.16	43, 55, 70	70	0.2–6.4	3^e^
2-Methylbutyl acetate	10.26	43, 55, 70	70	0.3–1.4	1.2 (in beer)^e^
2-Hexenal	10.28	41, 55, 57, 69	55	793.1–5902.1	49^d,e^
1-Hexanol	10.35	43, 56, 69	56	212.1–2025.7	200^e^
2-Heptanone (internal reference)	10.57	43, 58	58	50	70^e^
2-Heptanol	10.64	45, 55	55	253.1–526.5	100^e^
Methyl hexanoate	10.73	43, 74, 87	74	0.1–0.4	75 (in milk)^e^
(*E*,*E*)-2,4-Hexadienal	11.05	41, 67, 81, 96	81	23.3–214.0	36 (in oil)^e^
β-Myrcene	11.23	41, 69, 93	93	0.1–0.2	16.6^e^
2-Pentylfuran	11.32	53, 81, 82	81	0.1–2.9	4.8^e^
Ethyl hexanoate	11.45	43, 60, 88, 99	88	0.1–3.3	0.5^e^
Hexyl acetate	11.61	43, 55, 56, 61	56	0.5–5.5	40^e^
Octanal^a^	11.74	43, 56, 69, 84	84	-	0.52^d,e^
Linalool	12.62	41, 55, 71, 93	93	3.5–38.9	3.8^d,e^
Nonanal^a^	12.67	57, 70, 82, 98	57	-	4.25^d,e^
Hexyl butanoate	13.23	43, 56, 71, 89	89	0.1–2.1	-
2-Nonenal^a^	13.35	43, 55, 70, 83	70	-	0.5^e^
Decanal^a^	13.57	43, 57, 70, 82	82	-	3^d,e^
*Unknown terpene*^*a*^	13.85	93	-	-	-
Nerol	14.03	41, 69, 93	93	135.4^c^	-
Methyl geranate^a^	14.45	41, 69, 114	69	-	-

^a^Not quantified because no standard was available

^b^Concentration range [mg/kg]

^c^Only detected in one sample

^d^Adapted from [2]

^e^Adapted from [22]

During different metabolic pathways of plants, such as grapevines, manifold aroma compounds are produced from various precursors like fatty acids, carbohydrates, amino acids or carotenoids [23]. The majority of the aroma compounds detected in the analysed grape mash samples has already been found in previous studies on grape and wine aroma [24–26]. Moreover, most of the compounds have been detected in earlier examinations of grape mash by on-line NIR spectroscopy [16].

Due to different odour and taste thresholds, high absolute concentrations of aroma compounds do not necessarily imply high contributions to the overall aroma [2]. The contribution of an aroma compound to the overall aroma is indicated by the aroma value, which is calculated with Formula 1 [27].

1 $$\mathrm A\mathrm r\mathrm o\mathrm m\mathrm a\;\mathrm v\mathrm a\mathrm l\mathrm u\mathrm e=\frac{\mathrm C\mathrm o\mathrm n\mathrm c\mathrm e\mathrm n\mathrm t\mathrm r\mathrm a\mathrm t\mathrm i\mathrm o\mathrm n\;\mathrm o\mathrm f\;\mathrm t\mathrm h\mathrm e\;\mathrm a\mathrm r\mathrm o\mathrm m\mathrm a\;\mathrm c\mathrm o\mathrm m\mathrm p\mathrm o\mathrm u\mathrm n\mathrm d\left[\frac{\mathrm\mu\mathrm g}{\mathrm L}\right]}{\mathrm T\mathrm a\mathrm s\mathrm t\mathrm e\;\mathrm t\mathrm h\mathrm r\mathrm e\mathrm s\mathrm h\mathrm o\mathrm l\mathrm d\left[\frac{\mathrm{\mu g}}{\mathrm L}\right]}$$

**Fig. 1:**
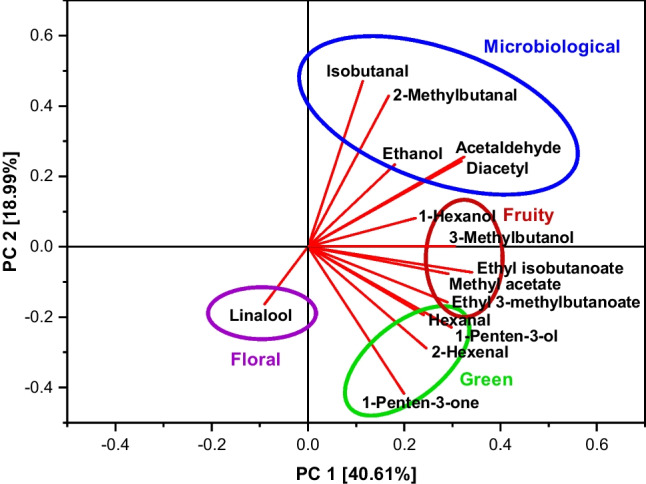
Loadings plot of the aroma compounds included in the calibration models for sensory attributes

To facilitate the development of NIR calibration models, only aroma compounds were considered, which reached or exceeded an aroma value of 10 in at least one analysed grape mash sample (Table 5). The selected aroma compounds were related to four different sensory attributes, which were chosen from the wine aroma wheels (“fruity,” “green,” “floral” and “microbiological”) [21]. At first, flavour descriptions were regarded to group the aroma compounds (Table 5). However, based on these descriptions, several compounds may be related to more than one sensory attribute (e.g. methyl acetate, 2-methylbutanal and 2-hexenal as both “fruity” and “green”). Moreover, the flavour descriptions are only given for a concentration of 30 ppm and different impressions may occur at varying concentrations. Because of the low concentrations of the compounds in the analysed samples, the classification was mainly based on a PCA, where a grouping of the aroma compounds already becomes apparent (Fig. 1). The only exception from the PCA-based grouping was made for 1-hexanol, which was related to the attribute “green/vegetative” due to its descriptions as “leaf alcohol” [2] and as contributing to “green” sensory characters [26]. The classification of the aroma compounds is shown in Table 6.

**Table 5:** Selected aroma compounds for calibration model development

Aroma compound	Range of aroma values	Flavour description at 30 ppm [20]
Acetaldehyde	147–1824	-
Ethanol	10–43	-
Methyl acetate	0–21	Green, ethereal, fruity, fresh, rum, whiskey-like
Isobutanal	19–735	-
Diacetyl	0–42	Sweet, buttery, creamy, milky
1-Penten-3-one	0–15	Pungent, ethereal, peppery, garlic, onion, fishy and mustard with a hot nuance
2-Methylbutanal	1–46	Green, fruity, musty with a berry nuance, musty, furfural and rummy, with nutty and cereal notes, caramel and fruity undernotes
1-Penten-3-ol	5–44	Green vegetable and fruity
Ethyl isobutanoate	0–100	Pungent, ethereal and fruity with a rum- and eggnog-like nuance; sweet, ethereal, fruity with a rum-like nuance
3-Methylbutanol	1–28	Fusel, fermented, fruity, banana, ethereal and cognac
Hexenal	10–51	Green, woody, vegetative, apple, grassy, citrus and orange with a fresh, lingering aftertaste
Ethyl 3-methylbutanoate	0–37	Fruity, sweet, estery and berry-like with a ripe, pulpy fruit nuance
2-Hexenal	16–120	Fruity, fresh green, herbal and vegetative, apple and melon with a slightly yeasty nuance
1-Hexanol	1–10	Green, fruity, apple-skin and oily
Linalool	0–10	Green, apple and pear with an oily, waxy slightly citrus note

**Table 6:** Grouping of the selected aroma compounds for calibration model development. *SD*, standard deviation; *PC1, PC2*, coefficients of the first two principal components of the extracted eigenvectors

Aroma compound	Concentration range [µg/L]	Average [µg/L]	SD [µg/L]	PC1	PC2	Sensory attribute
Acetaldehyde	3.2–40.1^b^	12.8^b^	8.3^b^	0.32460	0.25567	Microbiological
Ethanol	102.6–431.0^b^	329.4^b^	89.6^b^	0.18059	0.23393	Microbiological
Methyl acetate	18.6–1058.8	193.9	217.2^c^	0.29181	− 0.07682	Fruity
Isobutanal	7.4–293.9	78.9	66.5	0.11353	0.47020	Microbiological
Diacetyl^a^	29.3–224.1	51.4	48.0	0.31879	0.24331	Microbiological
1-Penten-3-one^a^	1.5–17.7	2.1	3.1^c^	0.19944	− 0.41716	Green/vegetative
2-Methylbutanal^a^	1.0–36.6	10.0	8.6	0.16772	0.42861	Microbiological
1-Penten-3-ol^a^	14.7–131.4	42.5	22.6	0.29808	− 0.22891	Green/vegetative
Ethyl isobutanoate^a^	0.2–2.3	0.3	0.6^c^	0.34054	− 0.07243	Fruity
3-Methylbutanol	74.9–2816.8	519.2	555.6	0.36021	0.00124	Fruity
Hexanal	2.0–10.8^b^	5.0^b^	1.9^b^	0.24045	− 0.19385	Green/vegetative
Ethyl 3-methylbutanoate	0.1–1.1	0.1	0.3^c^	0.28938	− 0.15741	Fruity
2-Hexenal	793.1–5902.1	1940.0	947.9	0.24562	− 0.28788	Green/vegetative
1-Hexanol^a^	212.1–2025.7	775.9	458.6	0.22333	0.08161	Green/vegetative
Linalool	3.5–38.9	2.3	7.2^c^	− 0.09004	− 0.16262	Floral

^a^Not included in the recalculated calibration models (see below)

^b^Concentration range/average/standard deviation [mg/L]

^c^The standard deviation exceeds the average value due to a strongly uneven distribution of the values within the concentration range

**Fig. 2:**
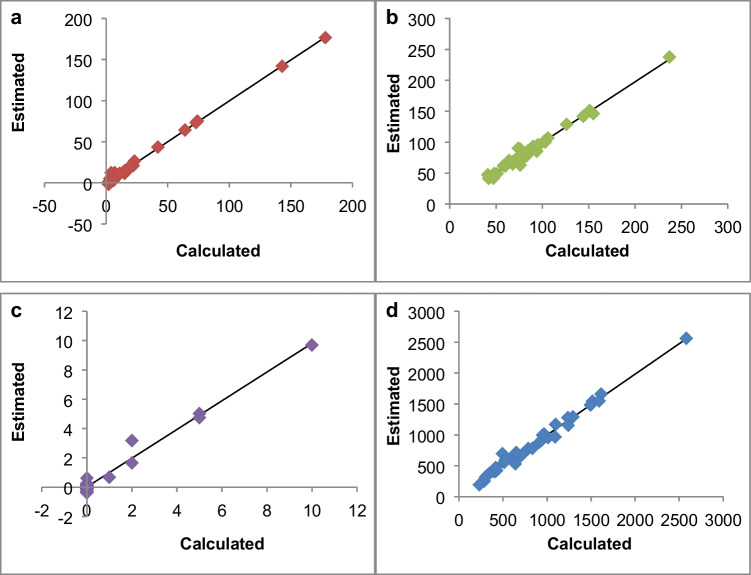
Correlations between the calculated and estimated summarised aroma values in grape mash for the sensory attributes **a** “fruity,” **b** “green/vegetative,” **c** “floral” and **d** “microbiological”

For every sample, the aroma values of the grouped aroma compounds were summed up and related to the corresponding NIR spectra to calculate calibration models for the four sensory attributes (Fig. 2).

The calibration models showed an excellent correlation between the aroma values calculated on the basis of the concentrations determined by GC–MS analysis and the estimated aroma values by NIR spectroscopy for all four sensory attributes (Table 7 and Fig. 2). A minimum value of 0.979 for the correlation coefficient of calibration (*R*^2^_C_) was reached for all calibration models while the SEC was relatively low (0.3 for “floral” to 60.7 for “microbiological”). The calibration models were validated by segmented cross-validation (5 segments). All four models reached a value of at least 0.943 for the correlation coefficient of cross-validation (*R*^2^_CV_). The SECV lies between 0.5 for “floral” and 106.7 for “microbiological,” which amounts to 3 to 5% of the range of values for all four models.

**Table 7:** Results of the calibration models for the sensory attributes (XLS regression, *N* = 36). *LV*, number of latent variables; *R*^*2*^_*C*_, correlation coefficient of calibration; *R*^*2*^_*CV*_, correlation coefficient of cross-validation; *SEC*, standard error of calibration; *SECV*, standard error of cross-validation; *MSC*, multiplicative scatter correction

Sensory attribute	Spectral pretreatment	LV	*R*^2^_C_	*R*^2^_CV_	SEC	SECV
Fruity	None	15	0.996	0.980	2.5	5.7
Green/vegetative	MSC	14	0.979	0.952	5.4	8.4
Floral	MSC	15	0.980	0.943	0.3	0.5
Microbiological	MSC	14	0.985	0.954	60.7	106.7

**Fig. 3:**
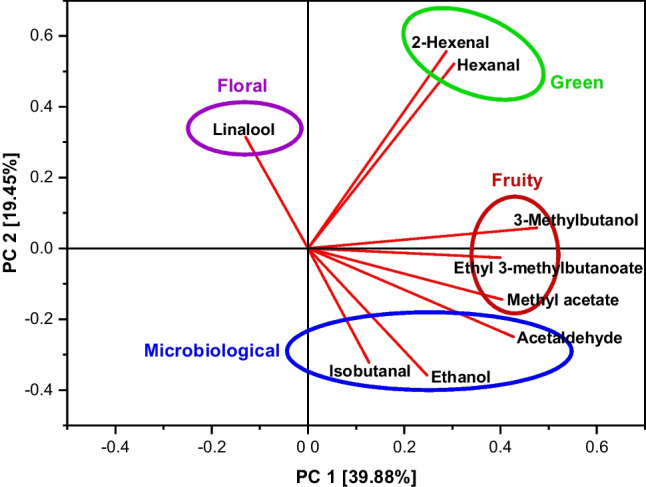
Loadings plot of the aroma compounds used in the grape model solutions for sensory analysis

To allow a better comparison of the results to the sensory evaluation, the calibration models were recalculated on the basis of the aroma compounds used for the grape model solutions (Table 6). The classification of the aroma compounds was not affected (Fig. 3). The results for the recalculated calibration models are given in Table 8. For the attribute “floral,” no recalculation was necessary.

**Table 8:** Results of the recalculated calibration models (XLS regression, *N* = 36). *LV*, number of latent variables; *R*^*2*^_*C*_, correlation coefficient of calibration; *R*^*2*^_*CV*_, correlation coefficient of cross-validation; *SEC*, standard error of calibration; *SECV*, standard error of cross-validation; *MSC*, multiplicative scatter correction

Sensory attribute	Spectral pretreatment	LV	*R*^2^_C_	*R*^2^_CV_	SEC	SECV
Fruity	MSC	13	0.986	0.965	2.1	3.6
Green/vegetative	MSC	14	0.976	0.917	4.5	8.7
Microbiological	MSC	14	0.984	0.951	60.5	107.2

The calibration models showed very high prediction accuracies for all four sensory attributes. No comparable studies were found for grapes, but earlier prediction studies of sensory attributes by NIR spectroscopy have been done for various wines. Good results were obtained for “fruity” attributes in red and white wines from two seasons (*R*_C_ = 0.90/0.85; *R*^2^_C_ = 0.81/0.73 and *R*_CV_ = 0.88/0.77; *R*^2^_CV_ = 0.77/0.59 for “fruity” in red wines, *R*_C_ = 0.91; *R*^2^_C_ = 0.83 and *R*_CV_ = 0.82; *R*^2^_CV_ = 0.67 for “tropical fruity” in white wines and *R*_C_ = 0.93/0.86; *R*^2^_C_ = 0.86/0.74 and *R*_CV_ = 0.77/0.83; *R*^2^_CV_ = 0.59/0.69 for “citrus fruity” in white wines) [10]. The attributes “floral” and “green” were examined in another study on Riesling wines. Visible and NIR spectroscopy showed a high correlation for “floral” (*R* = 0.71; *R*^2^ = 0.50 in cross-validation), but a low correlation for “green” (*R* = 0.38; *R*^2^ = 0.14 in cross-validation). Both values could be improved by additional use of MS-electronic nose (*R* = 0.73; *R*^2^ = 0.53 in cross-validation for “floral” and *R* = 0.45; *R*^2^ = 0.20 in cross-validation for “green”) [8]. Both studies did not examine possible correlations between volatile compounds and sensory attributes. Relationships between volatile organic compounds and sensory modalities, such as various gustatory and olfactory modalities, were examined in a study on a novel functional ice cream enriched with grape pulp and skins; however, NIR spectroscopy was not included in this study [28]. Analysis of volatile aroma compounds by GC–MS was performed under similar conditions to those in our study, except the use of solid phase microextraction (SPME). However, all aroma compounds determined in the ice cream mixtures were included in the calculation without consideration of their contribution to the overall aroma due to their odour and taste thresholds. In our study, higher prediction accuracies were obtained for all examined sensory attributes than in the studies carried out with wines. However, the dataset, on which the calculations are based, contains only a limited number of samples. Therefore, the models are probably not very robust yet and need to be extended with further samples, which should consider various aspects influencing the aroma of grapes (e.g. grape variety or growing area). Especially, the calibration models for the attributes “fruity” and “floral” show a very uneven distribution of the samples within the range of values and further work should focus on generating more data in the upper range. Furthermore, it has to be considered that the calibration models developed in this study are based on highly simplified reference values. Only aroma compounds were included in the calculation, which reached or exceeded an aroma value of 10 or more in at least one analysed grape mash sample. Aroma, especially in fruits, usually consists of a large number of compounds, which can cause different sensory impressions in their pure form [1]. Moreover, the classification of the aroma compounds is an important aspect. With an increasing number of compounds, PCA may only allow limited conclusions on how to classify the aroma compounds properly. The aroma descriptions of some compounds include a large number of different terms, so it may not be possible to relate the aroma compound to a single sensory attribute based on the description. Additionally, the concentration of the aroma compounds plays an important role, for instance acetaldehyde is perceived as fruity at low concentrations, but as pungent at high concentrations [2]. Even though the overall aroma undergoes extensive changes during fermentation and ageing processes, which can produce different wine flavours depending on the methods, the characteristics of the wine flavour for the grape variety usually remain recognisable [2]. The evaluation of sensory attributes enables an objective comparison of the delivered grapes according to their aroma quality as an additional aspect to usual quality parameters. For instance, the intensity of desired flavour nuances can be compared for delivered grapes from the same variety. Moreover, the determination of undesired attributes, such as “microbiological,” may be helpful for the decision about the treatment of the grapes, such as special treatment before winemaking (e.g. warming of the grape mash) or even not to use these grapes for winemaking.

### Sensory evaluation of grape model solutions

**Fig. 4:**
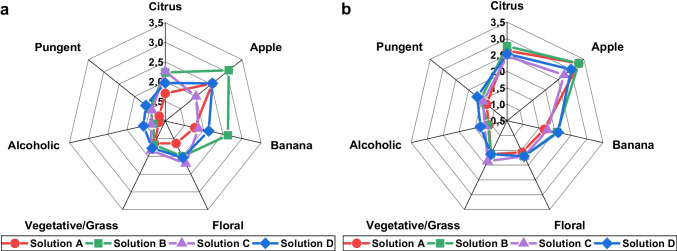
Averaged scores of the sensory attributes for odour (**a**) and taste (**b**) of the grape model solutions given by the members (*n* = 30) in the sensory evaluation

Odour and taste intensity of seven sensory attributes were judged in four different grape model solutions. The results are displayed in Fig. 4.

Regarding the odour, the scores for all sensory attributes were low in solution A, except for “apple.” This odour impression is probably caused by ethyl 3-methylbutanoate, which has a “fruity/apple” aroma, and 2-hexenal, which is described as not only “green” but also “fruity” and “apple” [2, 20]. Solution B got the highest scores for the attribute “apple” among all solutions, which may be explained by the fact that solution B also contained ethyl 3-methylbutanoate plus the highest amount of 2-hexenal among all solutions. Furthermore, the attribute “banana” was rated the highest in solution B, probably due to the high amount of 3-methylbutanol, whose aroma description includes “fruity” and “banana” [20]. Solution C got the highest value for the attribute “floral.” This may be explained by the fact that terpenoids tend to have floral aromas [2] and solution C is the only solution containing linalool. The attributes “alcoholic” and “pungent” were rated the highest in solution D, which contained the highest amounts of acetaldehyde, ethanol and isobutanal. All of these compounds were related to the attribute “microbiological” for the calculation of NIR calibration models (see above). Acetaldehyde is described as “pungent at high concentrations,” while the description of isobutanal includes “overripe” and “fermented” [2]. The results from tasting showed considerably smaller differences between the scores for the single solutions for all attributes and partly differed from the ones for sniffing. As with the scores for odour, solution D also received the highest values for the attributes “alcoholic” and “pungent” for taste. In contrast, the attribute “floral” was not rated higher for solution C than for the other solutions. While the odour of linalool is characterised as “floral/rose/woody,” its taste is described as “citrus” or “floral/green” [2], which may explain the difference between the scores. According to that, solution C was rated the highest for the attribute “green.” Particularly noticeable are the high values given for the attributes “citrus” and “apple” for all four solutions. During sniffing, only volatile compounds are sensed, while during tasting, the perception is also influenced by non-volatiles. The model solutions contain sugars and tartaric acid, which may have influenced the judgement considerably [2, 29]. The sweetness caused by the sugars may be associated with the attribute “apple,” while the sourness caused by the tartaric acid may be related to the attribute “citrus” or also “apple,” which could be the reason for the high scores given to these attributes during tasting.

**Fig. 5:**
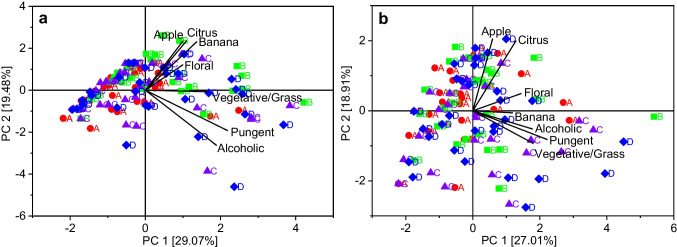
Principal component analysis score and loading plots for the results from the sensory evaluation of odour (**a**) and taste (**b**) in four different grape model solutions

In a one-way ANOVA (solution), statistically significant differences (*p* < 0.05) could only be observed between the solutions B and C for the attribute “apple” and between the solutions A and B as well as the solutions B and C for the attribute “banana” for the odour. For the taste of the solutions, no statistically significant differences (*p* < 0.05) occurred between the solutions for all attributes. PCA was performed on the results from the sensory evaluation for both odour and taste to examine possible grouping of the given scores related to the four different model solutions. The first two PCs can only explain 48.55% and 45.92% of the total variance of the given scores for odour and taste of the solutions. No separation was observed between the given scores according to the corresponding model solutions (Fig. 5). The given scores show a large variation for all four solutions and overlap almost completely. Regarding the odour of the solutions, solution B was slightly more influenced by the variables “citrus,” “apple,” “banana” and “floral” while the variables “alcoholic” and “pungent” had a slightly higher effect on the solutions C and D. Solution B contains ethyl 3-methylbutanoate and the highest amount of 2-hexenal, which are both described to have an “apple” odour and therefore may explain the effect of these variable. The effect of the variables “alcoholic” and “pungent” on solution D may result from the fact that this solution contains the highest concentrations of ethanol and acetaldehyde. However, solution C contains the lowest concentrations of both compounds, which indicates that the slight shift is probably caused by the large distribution of the results instead of the composition of the model solutions.

The sensory evaluation of grape model solutions showed diverging results (Figs. 4 and 5). On the one hand, differences between the average scores for the sensory attributes could be observed, which were higher for odour than for taste of the solutions. High values for sensory attributes could usually be explained by the concentrations of certain aroma compounds in the corresponding solution. On the other hand, statistical evaluation of the results showed that the given scores could not be grouped according to the different model solutions. Compared to the large variation of the single scores given to the sensory attributes for the various model solutions, NIR calibration models show very high prediction accuracies, which makes sensory evaluation seem less reliable. However, it has to be considered that the different sample matrices may have influenced the results, which complicates the comparison. Sensory evaluation has been carried out before for table grapes; however, regarding grape aroma, only the attributes “sweetness,” “sourness” and “flavour” were evaluated instead of single flavour nuances [30]. In another study, sensory evaluation of grape berries was carried out, which showed limited possibilities of predicting wine sensory attributes [31]. In contrast, our study only focussed on specific flavour nuances in grapes and no wine was produced for comparison. The participants in the sensory evaluation had different experience in tasting, which probably explains the large variety of the results. However, the average of the results from sensory evaluation often shows good reproducibility [27]. Additionally, the participation of both trained and untrained testers is probably more adequate, because winegrowers tasting grapes in the vineyard are not necessarily trained in sensory analysis and their personal preferences may also lead to biased results. Therefore, our approach may even be slightly closer to real grape tasting conditions. Furthermore, sensory perception is influenced by numerous factors, such as environment and stress, which can also affect the results of trained panellists. Due to the use of model solutions instead of grapes, the results for odour are specifically based on the selected volatile compounds, while the results for taste were also influenced by the addition of sugars and acid. The sugar/acid ratio plays an important role for the consumers’ acceptance of grapes [29] and was equal in all four model solutions. However, the overall impression, especially the taste of grapes, is influenced by further compounds, such as tannins [2], which were not considered in the model solutions. Therefore, the results from this study should be compared to those from a sensory evaluation with grapes. However, flavour may be influenced by colour, because darker colours can pretend higher flavour intensities, or by texture due to varying release of flavour active compounds [32]. Appearance, colour and texture can also provide information about grape quality before tasting, while model solutions may enable a more objective evaluation by judging solely the aroma. Beyond that, instrumental analysis, such as NIR spectroscopy, allows an objective evaluation by excluding the influence of personal impressions and preferences, but requires extensive further research on the contribution of the single aroma compounds to the different sensory attributes. Although sensory methods cannot be totally replaced by instrumental analysis, additional objective measurement is beneficial to increase the reliability of grape quality evaluation.

## Conclusion

Aroma compounds in grape mash have been determined under conditions similar to those in the mouth. NIR calibration models have been developed to determine sensory attributes in winemaking grapes upon receival at the winery based on grouped aroma compounds. The results were compared to those from sensory evaluation. The calibration models for the sensory attributes “fruity,” “green,” “floral” and “microbiological” showed very high prediction accuracies (*R*^2^_C_ ≥ 0.976). However, the models were based on a limited number of samples and the dataset should be extended to increase robustness of the models. Furthermore, the criteria on which the selection of the aroma compounds for the single attributes is based may need further evaluation. The given scores from the sensory evaluation of grape model solutions showed large variation, probably caused by the different tasting experience of the participants. No separation according to the different solutions was possible, however, averaged values of the given scores showed differences between the model solutions for most of the sensory attributes. NIR spectroscopy showed more promising results than sensory evaluation and may provide an alternative for evaluating sensory attributes, but extensive further research is required. Sensory evaluation cannot be totally replaced by instrumental analysis, however, on-line NIR spectroscopy can be used for an additional and more objective evaluation. For instance, evaluation of sensory attributes in grapes may enable the comparison of grapes from the same variety regarding the concentration of aroma compounds, which are responsible for desired flavour nuances of the resulting wine (e.g. terpenes) as an additional quality aspect. Further studies will show the potential of the method, also with regard to the inclusion of datasets from different years and growing regions.

## Data Availability

The datasets generated and/or analysed during the study are available upon reasonable request. For several datasets, the permission of the project partners may be required.
